# Preoperative Diagnosis of an Esophageal Duplication Cyst by Endoscopic Ultrasound Examination

**DOI:** 10.3390/diagnostics15091107

**Published:** 2025-04-27

**Authors:** Akane Shimakura, Kosuke Takahashi, Eisuke Ozawa, Hisamitsu Miyaaki

**Affiliations:** Department of Gastroenterology and Hepatology, Nagasaki University Graduate School of Biomedical Sciences, 1-7-1 Sakamoto, Nagasaki 852-8501, Japan

**Keywords:** esophageal cyst, esophageal neoplasms, esophagoscopy

## Abstract

A 78-year-old woman was referred to our hospital for close examination of an extramural submucosal tumor in the gastroesophageal region, suspected based on an imaging test performed for a chief complaint of epicardial pain while eating. Contrast-enhanced computed tomography revealed a 3 cm sized mass with well-defined margins and a homogeneous interior near the gastroesophageal junction. Endoscopic ultrasonography (EUS) revealed a large (28 mm) unilocular cystic lesion with a heterogeneous hypoechoic internal structure. The cyst wall was layered with a hypoechoic layer that appeared to be muscular and continuous with the external longitudinal muscle of the esophagus. Based on the EUS findings, an esophageal duplication cyst was diagnosed. Cystectomy was performed because the patient was symptomatic. Pathological examination revealed that the specimen was covered with columnar and pseudostratified ciliated epithelium without atypia and that the cyst wall comprised two layers of smooth muscle. No cartilaginous tissue was present, which is consistent with esophageal duplication cysts. Retrospectively, the EUS findings were consistent with the pathological findings.

**Figure 1:**
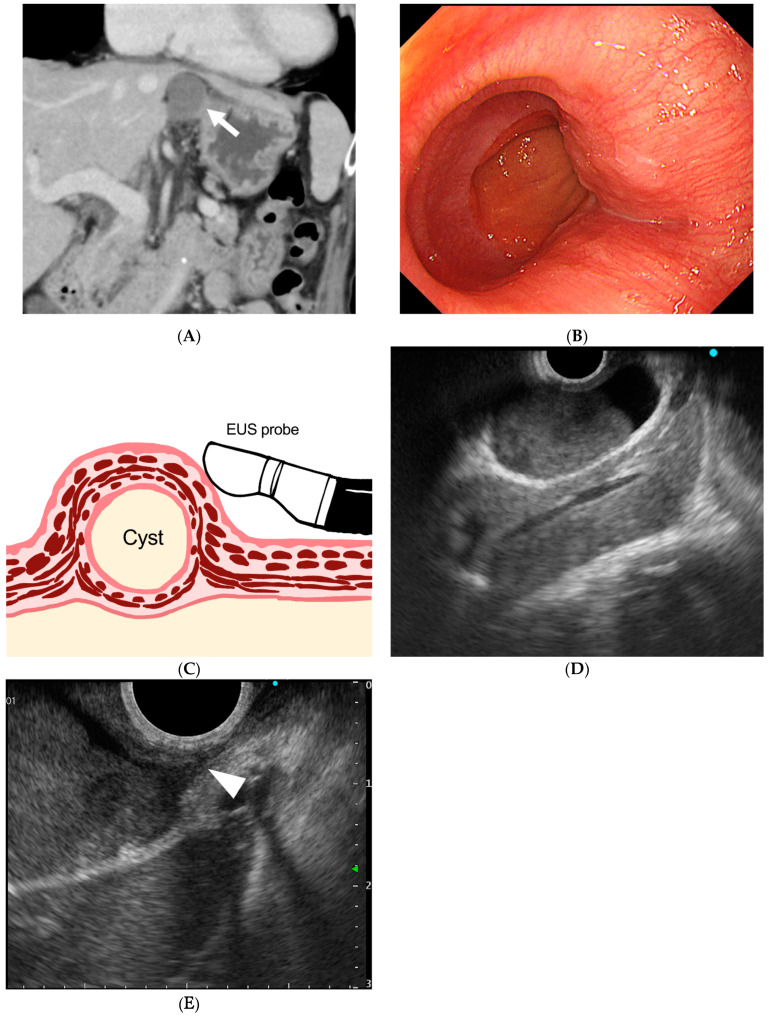
(**A**) Coronal section of contrast-enhanced computed tomography (CT). A 3 cm homogeneous mass with well-defined margins and a uniform interior near the gastroesophageal junction with a median Hounsfield unit value of 42.1, which was higher than the water density. (**B**) Endoscopic view of the esophagogastric junction reveals a 3 cm mass with normal surface mucosa and a gently rising mass on the mouth side of the esophagogastric junction. (**C**) Schematic diagram of esophageal duplication cyst and EUS probe. The cyst and the attachment site to the esophageal wall were observed using EUS. (**D**) Endoscopic ultrasonographic image. A 28 mm unilocular cystic lesion is visualized, with internal heterogeneous hypoechoic components suggestive of debris. Endoscopic ultrasonography (EUS) was performed using a curved linear echoendoscope (GF-UCT260; Olympus Corporation, Tokyo, Japan) connected to an ultrasound scanning system (EU-ME2; Olympus Corporation, Tokyo, Japan). (**E**) Magnified image of the area where the cyst wall is in close proximity to the esophageal wall. The cyst wall shows a layered structure, and the hypoechoic layer, presumed to be muscular, is partially continuous with the external longitudinal muscle of the esophagus (white arrow). Esophageal duplication cysts are characterized by cystic structures that possess a two-layered muscular wall similar to that of the esophagus and are attached to the esophageal wall [1]. Most cases present as unilocular lesions containing serous fluid, although debris may also be observed [2]. Differential diagnoses include bronchogenic cysts, gastrointestinal stromal tumors, neurogenic tumors, lymphangiomas, and pseudocysts. On EUS, key diagnostic features include continuity with the esophageal muscularis propria, a layered cyst wall, and a fluid-filled internal component. The absence of cartilage further supports the diagnosis of an esophageal duplication cyst. In the present case, imaging studies, including EUS, confirmed the cystic nature of the lesion, demonstrated a double-layered cyst wall, and revealed its attachment to the native esophageal muscle layer. These findings contributed to an accurate preoperative diagnosis.

**Figure 2:**
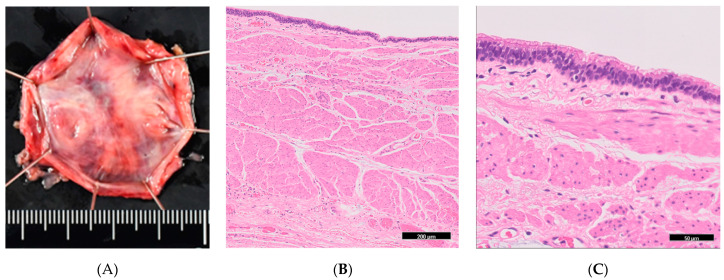
(**A**) Gross image of the resected specimen. (**B**) Pathological image with hematoxylin and eosin (HE) stain. The cyst wall shows a two-layered structure with an inner ring and an outer longitudinal muscle. (**C**) A highly magnified image of (**B**). The cyst wall is composed of columnar epithelium and multi-layered ciliated epithelium without atypia.

## Data Availability

No new data were created or analyzed in this study. Data sharing is not applicable to this article.

## Abbreviations

The following abbreviations are used in this manuscript:

CT	Computed tomography
EUS	Endoscopic ultrasound
MRI	Magnetic resonance imaging
